# What is full capacity protocol, and how is it implemented successfully?

**DOI:** 10.1186/s13012-019-0925-z

**Published:** 2019-07-18

**Authors:** Amir Alishahi Tabriz, Sarah A. Birken, Christopher M. Shea, Bruce J. Fried, Peter Viccellio

**Affiliations:** 1 Division of Pharmaceutical Outcomes and Policy, UNC Eshelman School of Pharmacy, University of North Carolina at Chapel Hill, 016 Beard Hall, 301 Pharmacy Lane, Chapel Hill, NC 27599-7355 USA; 2 Department of Health Policy and Management, Gillings School of Global Public Health, University of North Carolina at Chapel Hill, 1103E McGavran-Greenberg, CB #7411, Chapel Hill, NC 27599-7411 USA; 3 Department of Emergency Medicine, Stony Brook University, Health Sciences Center, Level 4 - Room 080, SUNY at Stony Brook, Stony Brook, NY 11794-8350 USA

**Keywords:** Emergency department crowding, Full capacity protocol, Intervention core components, Consolidated Framework of Implementation Research (CFIR), Emergency department management, Adaptation framework, Patient flow management, Hospital operations

## Abstract

**Background:**

Full capacity protocol (FCP) is an internationally recognized intervention designed to address emergency department (ED) crowding. Despite FCP international recognition and positive effects on hospital performance measures, many hospitals, even the most crowded ones, have not implemented FCP. We conducted this study to identify the core components of FCP, explore the key barriers and facilitators associated with the FCP implementation, and provide practical recommendations on how to overcome those barriers.

**Methods:**

To identify the core components of FCP, we used a non-experimental approach. We conducted semi-structured interviews with key informants (e.g., division chiefs, medical directors) involved in the implementation of FCP. We used the Consolidated Framework for Implementation Research (CFIR) to guide data collection and analysis. We used a template analysis approach to determine the relevance of the CFIR constructs to implementing the FCP. We analyzed the responses to the interview questions about FCP definition and FCP key principles, compared different hospitals’ FCP official documents, and consulted with the original FCP developer. We then used an adaptation framework to categorize the core components of FCP into three main groups. Finally, we summarized practical recommendations for each barrier based on information provided by the interviewees.

**Results:**

A total of 32 interviews were conducted. We observed that FCP has evolved from the idea of transferring boarded patients from ED hallways to inpatient hallways to a practical hospital-wide intervention with several components and multiple levels. The key determinant of successful FCP implementation was collaboration with inpatient nursing staff, as they were often reluctant to have patients boarded in inpatient hallways. Other determinants of successful FCP implementation were reaching consensus about the criteria for activation of each FCP level and actions in each FCP level, modifying the electronic health records system, restructuring the inpatient units to have adequate staffing and resources, complying with external regulations and policies such as fire marshal guidelines, and gaining hospital leaders’ support.

**Conclusions:**

The key determinant in implementing FCP is creating a supportive and cooperative hospital culture and encouraging key stakeholders, including inpatient nursing staff, to acknowledge that crowding is a hospital-wide problem that requires a hospital-wide response.

**Electronic supplementary material:**

The online version of this article (10.1186/s13012-019-0925-z) contains supplementary material, which is available to authorized users.

## Background

Emergency department (ED) crowding occurs when the need for emergency services exceeds the availability of ED resources [1]. While ED crowding is a complex issue caused by many extrinsic and intrinsic factors [2, 3], research has shown that the primary cause of ED crowding is boarded patients at the ED [2, 4]. “Boarded patients” refer to patients who remain in the ED after having been admitted to the hospital but are unable to be physically transferred to an inpatient unit because there are no available inpatient beds [5].

To deal with the practice of boarding patients, the American College of Emergency Physicians (ACEP) established a task force to develop a list of low-cost, high-impact solutions [6]. One of the key solutions proposed by ACEP is full capacity protocol (FCP). FCP suggests that when a patient requires admission to an inpatient unit from the ED and that unit cannot accommodate the patient due to lack of available beds, the patient will be admitted to the next most appropriate bed. In the event that appropriate hospital bed utilization has been maximized, selected admitted patients boarding in the ED are transferred to hallways in the inpatient units instead of boarding in ED hallways (i.e., inpatient boarding). While those patients are not physically in a room, they are able to receive care from inpatient physicians and nurse specialists, enabling ED providers to continue serving new ED patients [7, 8].

While the impacts of FCP on patients who are boarded at inpatient units have not been fully studied [9], several studies have shown that FCP has been associated with decreased ED length of stay [10], lower waiting time [11], fewer patients leaving the ED without been seen [10], lower patient mortality [7], higher operating revenues [12], and higher patient satisfaction [8]. Despite these favorable findings, FCP has not been widely implemented in many hospitals, even in hospitals experiencing extensive crowding [13]. There are multiple reasons why FCP has not been widely implemented including lack of a standard definition of FCP [9], barriers to FCP adoption [14], and lack of research on FCP implementation barriers and implementation strategies. To address this knowledge gap, research is needed to specify FCP core components and adaptable periphery (i.e., adaptable elements and structures, related to the FCP and hospitals that have adopted FCP) [15–17] and identify the key determinants of FCP adoption and implementation.

The objectives of this study were to identify the key components of FCP and the adaptable periphery and explore the key barriers and facilitators associated with the FCP adoption and implementation. Findings from this study could provide information helpful to medical centers on FCP design and development of strategies for FCP adoption and implementation.

## Methods

### Study design

This is a qualitative study utilizing in-depth, semi-structured one-on-one interviews with key individuals involved in the adoption and implementation of FCP.

### Selection of participants

Thirty-two hospitals were included in this study, 24 of which successfully adopted FCP and eight hospitals did not adopt FCP (i.e., non-adopters). The sample for this study consisted of 32 key representatives of those hospitals and ED operations representing a variety of roles (Table 1). Interviewing representatives of both adopters and non-adopters provided us with the opportunity to compare views on ED crowding and the approaches considered to address this problem. Ultimately, it enabled us to address the question of why hospitals choose to adopt or not adopt the intervention. Non-adopter hospitals in our sample are comparable with adopter hospitals on key characteristics including safety net status, trauma level, teaching status, region, and size.

**Table 1:** Staff roles of ED and hospital representatives (*n* = 32)

Representative role	*N*
Chief Nursing Officer	5
Nurse Manager	3
Chief of Emergency Medicine	2
Vice Chair of the ED	1
ED Medical Director	3
Associate Medical Director	2
Medical Director	13
Director of Operations	3

We used two methods to identify and recruit participants. First, we sent a recruitment email to all 231 the US hospitals with an ED affiliated with an academic medical center. We defined an academic affiliation as EDs serving as the primary site of an emergency residency program according to the Accreditation Council for Graduate Medical Education. We sent an email to non-responders up to three times at 1 and 3 weeks after the initial email. Second, we used a snowball sampling technique [18] in which we asked initial respondents to refer us to additional potential informants. We continued recruiting respondents until data saturation (no new information provided) was achieved [19].

### Theoretical framework

We employed the Consolidated Framework for Implementation Research (CFIR) [17]. CFIR provides a menu of domains and constructs that have been associated with effective implementation and can be used as a practical guide for systematically assessing factors related to implementing an intervention. Using CFIR allowed us to develop a clear picture of the complex, multi-layered nature of the implementation process. It does so by identifying the factors necessary for successful implementation, which in turn enabled us to evaluate the comprehensiveness of hospitals’ implementation strategies [17, 19].

We identified four CFIR domains (intervention characteristics, inner setting, outer setting, and process) and eight constructs (relative advantage, structural characteristics, culture, leadership engagement, access to knowledge and information, implementation climate, key stakeholders, and external policy and incentives) that were best aligned with our research aims and helped us to identify the potential barriers and facilitators to FCP adoption and implementation.

The core components are defined as the necessary actions and principles considered essential to successfully implement an evidence-based intervention (EBI) [15]. Core components generally include two parts: the essential principles necessary to produce desired outcomes (core content components) and EBI activities (core implementation components) [15, 20]. Principles describe the function of an EBI component (i.e., what are the EBI content components and why they matter), while activities indicate form (i.e., who is doing what, when, and where). In this study, we focus on the principles.

### Data collection

We developed a semi-structured interview guide based on CFIR (Additional file 1). The interview contained 15 open-ended questions covering the four preselected CFIR domains. We also asked respondents to explain their definition of FCP including its key principles and to share their related documents and official policies.

We conducted two pilot interviews to ensure clarity and minimize interview length and repetitiveness. We conducted interviews between October 2017 and February 2018 and each interview took between 35 and 55 min. We audio-recorded and transcribed verbatim all interviews. We obtained informed consent and guaranteed the confidentiality of all participants prior to the interview. The University of North Carolina at Chapel Hill Institutional Review Board approved this study (IRB # 16-2890).

### Data analysis

**Fig. 1:**
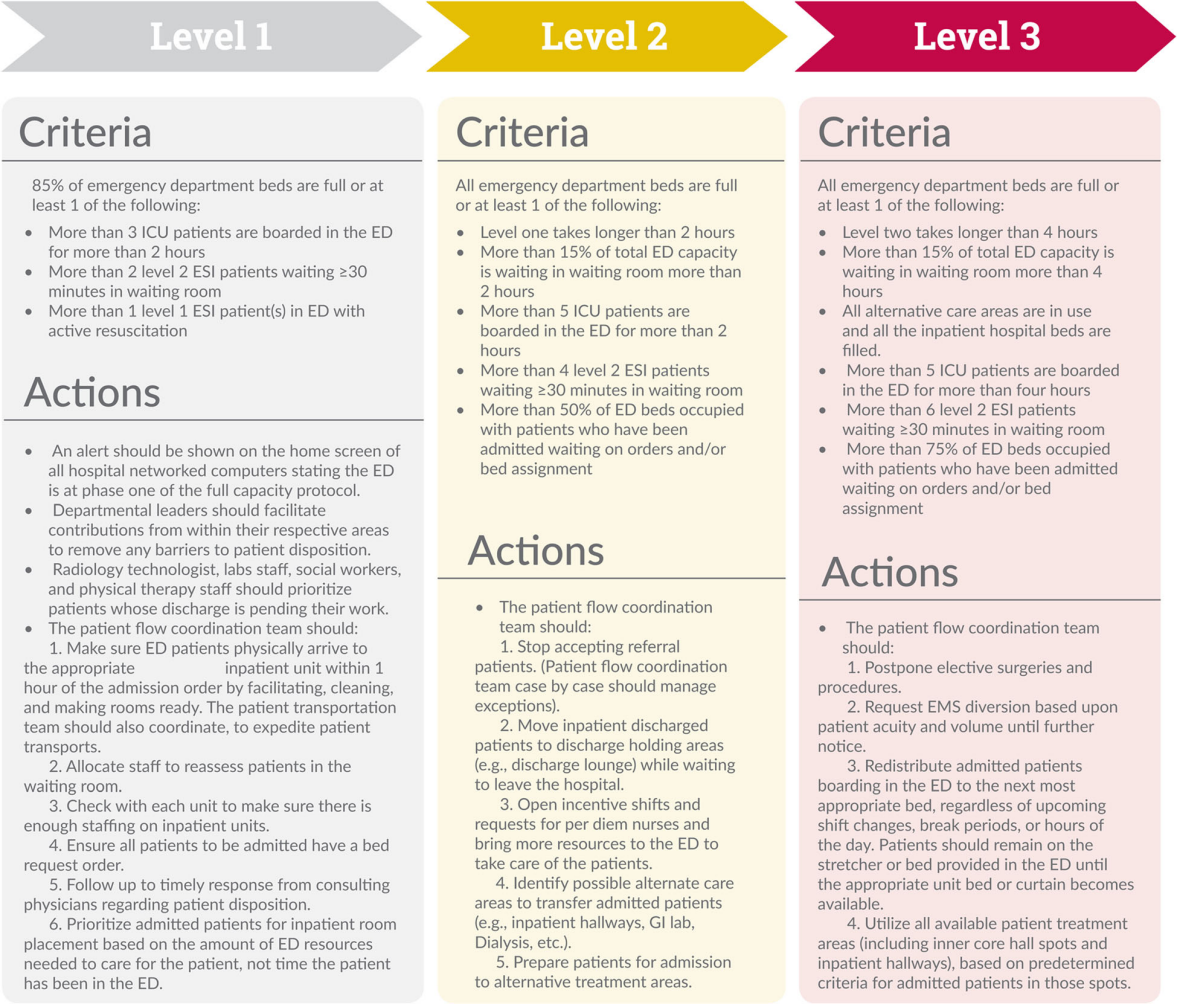
Hospital full capacity protocol

First, we reviewed FCPs from nine hospitals that successfully implemented FCP and agreed to share their protocols with us. We compared and identified the similarities and differences between those protocols, such as how they are structured and what the shared actions and elements are among them. Then we analyzed the responses to the interview questions about the essential functions and elements that define FCP at each hospital. We also consulted with content experts, including original FCP developers to understand the idea behind the FCP and its core elements. Finally, using perspectives of FCP original developers, input from informants who were directly involved with FCP implementation, and different hospitals’ protocols, we developed a broad overview of FCP (Fig. 1).

Second, we developed the FCP adaptation framework (Table 2). To do so, we used the Green/Yellow/Red (GYR) Light Adaptation guidance tool developed by CDC’s Division of Reproductive Health (DRH) [21, 22]. Among frameworks for adapting EBIs [23], we selected the GYR framework because it provides a practical way to define appropriate and inappropriate adaptations and to determine which changes could be made with minimal impact on effectiveness. Using the GYR light guidance tool, we categorized the components of the FCP into three main groups. The green light adaptation category is at the discretion of the hospital and does not affect fidelity or change the intent of the FCP. The yellow light adaptation category includes those components that should be adapted with caution and require expert consultation. The red light adaptation category includes those components that hospitals do not allow to change or ignore.

Third, we used responses to interview questions to explore the key determinants of FCP adoption and implementation, the main barriers hospitals faced, and how hospitals overcame those barriers. We used a template analysis approach [24], which is an approach to thematically analyze qualitative data using hierarchical coding. Template analysis provides a high degree of structure in the process of analyzing data, while also allowing for flexibility in summarizing and organizing identified themes [24]. To code the data, we used the CFIR NVivo project template (Additional file 2), which is pre-populated with CFIR construct codes. These codes facilitate organizing the data such that they can be used to develop case memos. We coded important passages in each interview into the related CFIR domains and constructs. We then compared codes within and across interviews to elucidate themes and selected quotations that were illustrative of each theme. We organized, labeled, and reported themes, using NVivo 10 software (QSR International).

After completing the analysis, to ensure the credibility of our findings, we sent the results to all participants for confirmation, congruence, validation, and approval (e.g., member checking) [25]. Ten participants responded and member checking did not result in any major changes or modifications.

## Results

### Characteristics of study subjects

We interviewed 32 representatives from 32 hospitals across 15 states. The role of each representative is presented in Table 1. All hospitals were urban, level 1 trauma and tertiary-care academically affiliated medical centers. All were safety net hospitals and experienced between 40,000 and 150,000 ED visits a year.

### Main results

#### FCP core components (i.e., what is FCP?)

We found hospitals defined FCP heterogeneously, each hospital adopting its own version of FCP. While hospitals used different variations of FCP, FCP was generally defined as a hospital-wide policy that includes different levels; each level containing several actions a hospital could take during crowding episodes (Fig. 1). Each morning, a multidisciplinary, hospital-wide patient flow coordination team had a morning census and safety briefing meeting to review the status of the hospital and see how many patients, admissions, and discharges were anticipated for that day. The patient flow coordination team typically included a nurse manager, medical director, director of clinical operations, and at least one person from the hospital executive level. Hospitals operated a routine flow until certain internally assigned sets of criteria were met and then a FCP level 1 was activated. If the patient flow coordination team, however, anticipated an increase in patient volume, such as a low discharge volume or high admit volume, the patient flow coordination team might preemptively activate the FCP level one to avoid an expected increase in throughput.

We designed our own version (FCP). We call it capacity protocol, and we have three levels. For each level, there is a series of criteria that we have set and at each level there are various things that happen. We also have the ED flow coordinator, which is a position that we created alongside with doing the capacity protocol. The flow coordinator has the capability to activate the protocol early if they recognize that things are soon going to reach a level two.

#### FCP adaptation framework

Each hospital created its own version of FCP to increase FCP compatibility with their hospital system. We observed that hospitals varied in the criteria for activation of each FCP level and the actions taken at each level. For example, we observed that some hospitals defined the activation criteria to ensure that patients are rarely transferred to an inpatient hallway. In contrast, some hospitals routinely transferred patients to the inpatient hallway. A participant described how they decided to select the criteria for activation of each level and associated actions:

There’s reluctance at the hospital administration level to put patients in the hallway upstairs, so that would happen, but very, very rarely, probably once or twice a year. So what would happen more often is pressure being placed on the upstairs people to get patients discharged or to move into a discharge lounge to wait for their ride.

To successfully implement FCP, it is necessary to balance fidelity and the core components of FCP, allowing for flexible adaptation to context-specific factors [26]. Using the Green/Yellow/Red Light Adaptation guidance tool [20, 21], we developed the FCP adaptation framework as guidance about components that can and cannot be altered from FCP. The FCP adaptation framework shown in Table 2 describes the types of adaptations that are safe (green) such as naming the protocol or the timing of the morning safety huddle, those that merit caution (yellow) such as adding other ED crowding interventions, and those that should be avoided (red) such as putting patients in areas that obstruct patient and staff flow.

**Table 2:** Full capacity protocol adaptation framework

Green	
Things that can be changed:	
•Name of the protocol (e.g., escalation policy)	
•Time of morning safety huddle	
•Incentives for participation	
•Format and wording of the protocol	
Yellow	
Things that can be changed/modify with caution:	
•Number of levels (generally 3 or 4 levels)	
•Activation triggers for each level	
•Actions in each level	
•Order of actions in each level	
•Add other ED crowding interventions (e.g., use of discharge lounges, surgical smoothing)	
•Generally aim to place no more than 1 to 2 patients on any one-inpatient hallway. Hospitals cautiously can change this to whatever is needed, depending on crowding situation, the physical environment on each inpatient unit, and available staff and resources in inpatient units.	
Red	
Things that cannot be changed/ignored:	
•Do not change the order of the levels (sequence)	
•Do not delete an entire level of the protocol	
•Place patients in areas with access to a bathroom	
•Place patients in areas that least obstruct flow	
•Do not transfer patients who are not eligible to transport to inpatient hallways including:	
1. Patients need intensive care unit (ICU) or cardiac care unit (CCU) bed	
2. Patients requiring negative pressure room	
3. Patients requiring 4 L or greater of oxygen	
4. Patients that require suctioning	
5. Patients with unstable vital sings	
6. Patients with Glasgow Coma Score < 15	
7. Mechanically ventilated patients	
8. Psychotic patients	
9. Patients that have diarrhea or are incontinent of stool	
10. Patients at immediate risk of seizures	
11. Patients with open wounds	
12. Patients at high risk of bleeding	
13. Children and patients who are 75 years and older	
14. Patients with recent high-risk coronary artery disease	
15. Patients with history of heart failure, stroke	
16. Patients with history of peripheral arterial disease	
17. Patients with chronic obstructive pulmonary disease	

### Determinants of FCP adoption and implementation sorted based on CFIR domains

#### Domain: intervention characteristics

##### Relative advantage

Relative advantage refers to the perception that an intervention would be beneficial to the organization compared to other interventions [17]. Participants recounted comparing FCP with other ED crowding interventions, such as expanding the ED, and described the advantages of FCP. For example, one participant indicated that FCP allowed hospitals to increase patient volume without increasing the length of stay. Additionally, some participants explained that FCP directly reduced boarding time and increased the number of nursing hours per patient at the ED. This allowed hospitals to place patients in a quiet area with an appropriate nurse and physician coverage. Participants also felt that implementing FCP was easier and less costly than other ED crowding interventions because it does not require as many resources and training for implementation.

We had a couple of other things that were already going in parallel to this, but it actually decreases our walkouts. It decreases our left without being seen because it facilitates our ability to see our emergency patients. It improves the entire hospital operations. Inpatient staff began to work with case management, they worked with social services to find ways to get people out of the hospital early.

In spite of these advantages, the participants mentioned some challenges. First, it was noted that FCP requires that changes need to be made to the electronic health records (EHR) system. No EHR had designated admission slots to identify patients boarded in a hallway. Some hospitals modified their EHR to include specific slots for those patients. Additionally, some hospitals adapted their EHR to incorporate FCP levels and add a banner on their EHR, which shows them the capacity level in real time:

The one sort of challenging part of it was we wanted Epic ... So we use Epic as our electronic medical record. We wanted them to be able to create these additional spots. So you had to have them create a virtual spot to put the patient into on the floor, so that way the nurses could chart and the doctors could write notes and et cetera, et cetera. So they had to get all that built in Epic.

Additional challenges include a lack of physical space and shortage of inpatient nurses to provide care for inpatient boarded patients. Implementing FCP may increase the workload of inpatient nurses. Some participants stated that in high-pressure work environments with limited resources, such as some inpatient units, transferring patients to the inpatient hallways could put patient safety at risk. Some institutions lacking the physical space to place beds in hallways use patient care areas not typically used during evenings and weekends for temporary boarding, such as perioperative holding areas. Respondents from some hospitals described a lack of staff, primarily inpatient nurses, as a key challenge:

We actually have physical inpatient beds that are just not staffed. So we, on any day of the week, have 30 or so physical beds that could be used for patients, but we don’t have nurses for them. So I don’t think that we wouldn’t need to even put people in the hallway. We could just put them in the beds that aren’t staffed.

#### Domain: inner setting

##### Structural characteristics

Structural characteristics concern the social and physical architecture, age, maturity, and size of an organization [17]. We observed that the size of the hospital system is a critical factor in FCP implementation. FCP is a hospital-wide intervention that requires collaboration and coordination between many departments throughout the hospital. The larger the hospital, the more difficult FCP is to coordinate:

You know our hospital system is like a city, so any change in that scale needs tons of paper works and meetings to get all the players on the board. We have a joke here that said passing a law in the Congress is easier than making a change here.

In addition, some representatives mentioned that they had to restructure their inpatient units to have adequate staffing and resources, such as central telemetry monitoring, privacy screens, a wireless call system, portable monitor/defibrillator and portable suction equipment, appropriate bathroom facilities for transferred patients, and other resources necessary to provide high-quality patient care.

##### Culture

Organization culture is defined as the norms, values, and basic assumptions guiding an organization [27]. Many interventions fail because organizations neglect to account for or change less tangible organizational assumptions, thinking, or culture [17]. Respondents expressed that an important challenge to FCP implementation is to communicate to key stakeholders that crowding is a hospital-wide problem that requires a hospital-wide response. Implementing FCP may require changing the culture of the entire hospital to ensure that the culture is supportive of this collaborative strategy.

People in our health system just seem to be too comfortable having patients wait for their care and we were not comfortable with that. So just trying to overcome that culture, both from an inpatient point-of-view as well as an emergency department point-of-view, to be honest, that’s a big challenge as well.

In addition, individuals in hospitals that did not adopt FCP usually perceived the ED crowding as solely an ED problem and unrelated to the entire system.

No, it (ED crowding) is not a hospital problem. The ED crowding because of the nature of the emergency department, the front door is always open. We cannot close the front door of our ER. The back door of our ER closes all the time. So, the ER has for years, been able to manage that accordion style flux and surge without really having any kind of offshoot, or any kind of mechanism to take the capacity. So, as it stands now, we’ve done so well at managing it, and we continue manage it without complaining or without making a big deal. It’s not a hospital problem, it’s solely an ER problem.

Respondents also reported that the long-term success of FCP depends on whether staff members are willing and able to change their behavior. Hospital staff members are unlikely to participate in the FCP implementation process if they think that FCP is designed simply to save money or ease the workload of ED personnel. However, if they understand that FCP’s main goal is to improve patient care, quality, and safety, they will be more likely to embrace the need for change because their beliefs and emotions will be engaged.

I mean I think we have a collaborative culture that’s very based around the patient and trying to do the right thing for the patient. I think there are good relations with the inpatient and administration. I don’t think we always necessarily agree on the best way to go about things, but I certainly think if we were able to make a compelling argument, then we may be able to make grounds on it.

##### Leadership engagement

In hospitals that adopted FCP, participants agreed that leadership engagement helped facilitate FCP adoption. Having hospital executives that are aware of the negative effects of ED crowding and supportive of addressing ED crowding at the hospital level is critical for FCP adoption. In addition, participants emphasized that FCP implementation requires a clear commitment by hospital leadership to overcome operational barriers across departments. The hospital senior executive team must visibly support the program. Hospital leadership and ED leadership should be aligned throughout the implementation process. Because implementing FCP may require substantial resources, participants believe that FCP cannot move forward without senior leadership support. One participant described the importance of leadership support:

You need complete support from the executives in the hospital, they have to be willing to be champions and truly eliminate barriers and you have to have a mechanism to develop relationships between the different departments and the staffs in the different departments, because if you don’t have those relationships and you haven’t built the groundwork and they haven’t felt involved in the process, then the process is going to hit a lot of resistance or may fail.

In contrast, representatives from hospitals that did not adopt FCP suggested that one of the main reasons that they did not adopt FCP was their inability to achieve consensus among members of the hospital leadership team about the importance of ED crowding and the need to address the issue at the hospital level. Specifically, participants indicated that having the support of the chief nursing officer (CNO) is necessary for FCP adoption; CNO opposition is a major barrier to creating the consensus required to adopt FCP.

Despite our efforts such as meetings, grand rounds with national leaders on this topic to advance the adoption of a full capacity protocol we have not been able to convince the hospital to do this. It was essentially blocked by our CNO, who was in place for years.

##### Access to knowledge and information

An important determinant in FCP implementation is having access to information about FCP. Through the recruitment stage of our study, we observed that even some ED chairs and program directors did not know whether their hospital had implemented FCP. We also found that hospitals typically do not have a formal education program or an online module to educate physicians and staff. However, some respondents reported using faculty meetings, nursing meetings, and emails to engage and educate key individuals involved with implementing the protocol:

I don’t think that there was a lot of training necessary on the ED and inpatient side of things. And really, it was just the training that was necessary with the proper selection of the patients who are going upstairs and that was for sort of the shift supervisor of the ED and that’s about it. I think it was just the persistence of being exposed to this multiple times and the repetition probably helped and just soaked into the culture.

##### Implementation climate

Implementation climate is defined as the capacity for change, receptivity of stakeholders, and the extent to which the use of the intervention will be treated, rewarded, and supported [17, 25]. Some participants indicated that they believed there were few incentives for or endorsements of FCP in their organization. Respondents reported that hospital leaders, staff, and inpatient nurses were often resistant to FCP, but accepted it simply because they did not believe they had any other choice. Despite low levels of receptivity, hospital leaders and other stakeholders viewed FCP implementation as a protocol that is beneficial to patients:

They (hospital leaders) have helped to create it. They wish they didn’t have to deal with it, so they don’t like it in that sense, but this takes up an enormous amount of their time and energy. I think, they like the process and are proud of it when they saw the diversion rates go down significantly.

#### Domain: outer setting

##### External policy and incentives

External policy and incentives refer to external factors that may affect the adoption of an intervention, such as governmental policy and regulations, external mandates and benchmark reporting, recommendations and guidelines, and financial incentives [17]. Participants indicated that external policies and incentives had a great impact on the FCP adoption process. Some hospitals, for example, were reluctant to adopt FCP due to fire marshal guidelines that prohibited putting patients into hallways.

There was a local hospital that’s one of our competitors did it, approximately a year ago. And they had the fire Marshall called in, in short order and it was shut down. So that has kind of remained an anecdote of why we can’t do this.

Nursing guidelines on patient/nurse ratio constituted another external policy that affected FCP adoption decisions. Specifically, hospitals that already faced nurse shortages were concerned that transferring patients to the inpatient hallway would violate patient/nurse ratio guidelines.

There are rules about nursing ratios on floors. When you’re over that, there will be a ton of pushback on that.

Finally, inter-hospital issues were identified that may affect the decision to adopt FCP. For example, it was noted that when one hospital stopped accepting patients and went on ambulance diversion, other hospitals in the area may experience a patient surplus and begin diverting ambulances, potentially causing ED crowding to spread throughout other hospitals in the community (i.e., a domino effect). One barrier to adopting interventions such as FCP is hospital leaders’ fear of negative consequences resulting from successfully addressing ED crowding. Being the only hospital in the region that accepts all the patients could lead to uninsured patients flooding into the hospital, which might financially damage the hospital.

One of the things that were brought up by the faculty and the nurses was that, when we were crowded it puts significant strain on them to accept more patients, especially ambulances. The walk-in patients will walk in, and there’s no way to deter them, but as long as we continue to be successful with that (FCP), we also accept all ambulance patients diverted from other hospitals. To some extent, we’re victims of our own success.

#### Domain: process

##### Key stakeholders

In addition to hospital leaders, there are two other key groups that must be on board to successfully implement FCP: nurse managers and patients. Noteworthy were participants’ comments about resistance from inpatient nurses. FCP requires a major cultural change and will likely increase nurse workload. Nurse managers play a key role in helping other staff understand why implementing FCP is important for ED patients. To garner the support of inpatient nurses, some nurse managers have met with different nursing teams to hear their concerns and use their input to improve FCP. Because there may be resistance from nurses on specific nursing units, generating support from nurse managers is essential. Absent their support, implementing FCP is doomed to fail:

So it was buy-in at the nursing leadership level that was very important and the most influential people in this actually were those nurse managers. Because it’s the nurse managers that need to go back to the nursing staff and say we are doing this for our patients. If they had gone back to their nurses and say listen to what they’re making us do now, putting patients in hallways, this would have never worked.

The ultimate goal of all EDs is to provide timely and high-quality care to patients. Participants emphasized that patients should be informed and engaged in advance of any major changes. Patients have to be taught why the change is occurring and how it may affect them. Participants stated that hospitals should identify opportunities to explain to patients the need for this strategy and that safety and quality are unaffected:

You can’t tell a patient in the ED, Oh, we’re sending you to your floor now. They are expecting a nice bed and a room and they get a bed in the hallway. They have to know where they’re going, so those are all training components for us there*.*

## Discussion

Despite a variety of ED crowding interventions [13, 28], hospitals continue to struggle with ED crowding and its consequences [29]. Hospital leaders may know which interventions they want to adopt but may lack knowledge about the essentials of implementation. FCP is no exception.

We found over the past 20 years that FCP has evolved from the idea of transferring boarded patients from ED hallways to inpatient hallways to a complex hospital-wide intervention with several components and multiple levels (Fig. 1). While inpatient boarding is still the main component of many FCPs, some hospitals have adopted FCP without inpatient boarding. In contrast, some hospitals decided to simply transfer ED-boarded patients to inpatient hallways without adopting the entire protocol.

Consistent with Back et al. [30], we found hospitals adapted FCP based on their local needs, gaps, principles, and hospital culture. However, reaching consensus about the criteria for activation of each level of FCP and actions in each level of FCP is challenging. For example, if hospitals choose less restrictive criteria for activation at each level, they often remain in FCP level 2 or 3. As a result, FCP may not be as effective, which could lead to a lack of political will for implementation or intervention fatigue. In contrast, if a hospital chooses criteria that are too restrictive, such as taking no measurable action until getting to FCP level 3, FCP will not be efficient and will become an inoperable policy instead of a practical policy for alleviating ED crowding. A better approach may be to develop a FCP based on hospital demand. Instead of putting a restricted cap on the number of patients in inpatient hallways, some hospitals used a demand-based approach and adjusted the criterion in real time based on the magnitude of the crowding situation, the physical environment on each inpatient unit, and available staff and resources in inpatient units.

We also identified the main barriers to FCP adoption and implementation. Fear of exacerbating ED crowding because of other nearby crowded EDs (i.e., domino effect), impacts of external regulations and policies (e.g., fire marshal regulations), lack of knowledge and information about FCP, limited resources, and lack of leadership support and commitment are among the most dominant barriers to FCP adoption and implementation. In Table 3, we summarize and present practical recommendations for each barrier based on our interviewees and analysis.

**Table 3:** Full capacity protocol implementation barriers and recommendations to overcome those barriers

Barrier	Recommendations
Inability to reach the consensus about the criteria for activation of and actions in each FCP level	•Collect and analyze operational data to create a predictive model and patient flow map. A predictive model and patient flow map gives a hospital the opportunity to appropriately plan resource allocation and prepare to address patient flow variability. Adapt the criteria for activation of each FCP level based on hospital unique flow variation [31] and identified hurdles.
Lack of knowledge and information about FCP	•Disseminate knowledge and train various stakeholders about the FCP. Hospital stakeholders should be aware of the protocol and their new operational responsibilities. For example, clearly defining that once an admitting physician has accepted a patient, that admitting physician is responsible for the patient throughout the admission, regardless of patient location.
	•Key staff members such as nurse managers should be trained to participate in FCP implementation. These trained staff members can then become champions and coaches for others in the hospital.
Limited resources	•Provide adequate staffing and resources to inpatient units. Some examples of resources necessary to successfully implement FCP may be central telemetry monitoring, privacy screens, a wireless call system, portable monitor/defibrillator, portable suction equipment, and appropriate bathroom facilities for transferred patients. Remember, “adequate staffing” is relative to the patient’s viewpoint. Moving a patient upstairs may yield less than optimal ratios; but it may improve patient satisfaction. Reorganize hospital resources (e.g., EHR modification), revise existing operational procedures, and/or create new structures in line with FCP.
Lack of leadership support and commitment	•Ask hospital leaders to personally visit the ED to view the crowding first-hand.
	•Tell a compelling story about what is going on with boarded patients and why they are not getting the care they need—how they are suffering because of crowding.
	•Change some of the verbiage when sending information to the executive team and across the hospital. Describe the problem as a hospital capacity issue instead of an ED crowding problem.
	•Take responsibility; do not blame other parts of the hospital.
	•Make crowding a priority for hospital leaders.
	•Present hospital leadership with studies that demonstrate financial opportunities lost due to crowding and ED profitability. Emphasize that crowding could indirectly damage the hospital by hurting the hospital’s reputation, increasing hospital length of stay, adversely affecting mortality and clinical outcomes, putting the hospital in danger of losing other certifications, and decreasing atient satisfaction. Many of these can affect CMS reimbursement.
	•Hospitals tend to abandon the entire idea of FCP because of concerns about placing patients in inpatient hallways. Try to include inpatient hallway placement in the protocol, but do not sacrifice the entire FCP for this component.
	•Outline the pros and cons of FCP over other known ED crowding interventions.
	•Remind leaders that adopting both FCP and other ED crowding interventions are not mutually exclusive. One option could be to implement a combination of ED crowding interventions based on the hospital culture, needs, and resources.
	•Do not oversell FCP. Be clear that it is not ‘the solution’ for crowding. Rather, FCP has been demonstrated to successfully reduce crowding.
	•Hospital leaders should provide a consistent message about adhering to the protocol by providing tangible assessment and appreciation. Hospital administration should offer a modest but visible reward program. Reward systems may include informal celebrations, small denomination gift certificates, and senior leadership personally thanking staff on the floor for their efforts.
Cultural resistance	•Explain the benefit of FCP to all providers, specifically inpatient nurses.
	•Involve all hospital members in FCP planning. Pre-implementation involvement helps reduce barriers to change by creating psychological ownership, promoting the dissemination of critical information, and encouraging employee feedback for fine-tuning the change during implementation.
	•Let key stakeholders know the appropriately assigned thresholds. For example, the higher level of FCP should rarely be activated (if ever).
Inpatient nursing resistance	•Listen to, acknowledge, and respect the concerns of nurses. Common concerns have been a lack of monitoring and threats to patient privacy and safety.
	•Emphasize that transferring patients to inpatient hallways is a last resort in dealing with crowding. The main purpose of FCP is for patients, not the ED. It is designed to optimize patient care in suboptimal circumstances.
	•People are more receptive to participate in change when they perceive potential for personal and organizational benefit after weighing the strengths and weaknesses of change [32].
	•Ask nurse managers to help you address the problem. Show them that their efforts will not only help patients but also improve the work environment for ED nurses by more evenly re-distributing the workload throughout the hospital.
	•Emphasize that FCP is not about room versus hallway; it is about which hallway.
	•Work with CNOs to create an environment in which the floor nurses “pull the patient up” rather than the ED nurses “pushing the patient to the floor.”
Concerns about domino effect	•Communicate with local organizations. Consider inviting competitors to observe your processes.
	•Host town halls with community hospitals to present your metrics and process improvements.
	•Educate local community hospital directors and nursing leadership about FCP. Ask them to join you.
External policies and regulations	•Before officially adopting FCP, address relevant regulatory guidelines that would have an impact on hallway boarding policies, such as those from the Joint Commission and the appropriate state regulatory bodies. It may be necessary to obtain approval from relevant regulatory bodies prior to FCP implementation. Joint Commission has not typically required prior approval, as long as fire safety regulations are addressed.
	•Work with the local fire marshal to determine how to safely implement the FCP. It is only through consulting with the fire marshal that one may determine how to overcome regulatory obstacles to inpatient hallway boarding.
	•Justify FCP to fire marshals with two key concepts. First, describe how patient safety concerns are equally as critical in the ED as in the inpatient setting. Both should be viewed as acceptable. Second, describe how the risk of keeping a patient in an ED hallway is much greater than transferring that patient for a short period of time to an inpatient hallway.
	•Conduct fire drills that involve transferring patients, how transfers are to be carried out, and actions to take in the event of a fire.

The results of this study suggest that inpatient nurses are often the most vocal opponents of inpatient boarding. Major concerns include patient safety, crowding in inpatient units, higher risk of cross-infection, and increased inpatient team workload. In addition, because the ED is an isolated unit in many hospitals, the consequences of inefficient patient flow may not be visible to individuals in other hospital units; crowding may simply not be perceived as a problem [33]. As a result, staff in an inpatient unit may consider only their own flows and may not recognize crowding as a hospital-wide issue capable of creating dysfunctions elsewhere in the organization [34]. A study by Pulliam et al. showed that inpatient nurses and those who have never worked in the ED are more likely than others to oppose inpatient boarding [14]. In contrast, inpatient nurses appreciate the specific skill set they bring to the bedside of a sick patient. For instance, an oncology nurse may readily support the idea that an oncology patient is better off in the hallway of an oncology unit than in the hallway of the ED. When it is clearly presented that the choice is not a hallway vs. a room, but a hallway in the ED vs. a hallway on the unit with better staffing ratios and appropriate nursing expertise, nursing is more likely to support the concept of the FCP.

Finally, we found that another key determinant of FCP adoption and implementation is the ability to change the hospital culture. Research has identified that culture is a notable predictor of implementation success and sustainability of change [35]. If providers do not understand the benefits of FCP, providers are likely to resist FCP implementation, particularly given the long-standing practice of leaving admitted patients in the ED. We found that in hospital systems where the dominant culture theme is “we always do it that way,” successful FCP implementation requires a combination of lower-level extensive participation and top management commitment [36, 37]. In line with Birken’s theory of the middle managers’ role in healthcare innovation implementation [38, 39], our findings suggest that hospitals should have a vice president or similar leadership position (e.g., patient flow coordinator) focused on patient flow to serve as a bridge between hospital leaders and ED physicians, mid-level providers, medical records staff, and front desk staff. A person who holds that position should be responsible for representing ED concerns at the executive level, engaging hospital leaders and nurse managers, and advocating for FCP with senior hospital leadership. In addition, this individual should ensure that front desk staff are provided with relevant information and resources and may need to mediate day-to-day activities and educate, monitor, and troubleshoot FCP implementation [30].

This study has several limitations. First, the responses of a selected group of hospitals’ representatives may not be representative of larger trends across the country. While qualitative studies are mainly about depth rather than breadth, we addressed this limitation by selecting representatives from different states. Second, we did not interview hospital leaders and inpatient nurses. Therefore, we are not fully aware of their concerns and thoughts about the crowding issue, inpatient boarding, and FCP implementation. It is possible that hospital executives have a different perspective on ED crowding and FCP implementation. The next step to understand FCP adoption and implementation would involve interviewing hospital administrators and inpatient nurses to explore their perceptions about ED crowding and thoughts about the feasibility and acceptability of FCP. Finally, in this study, we only focused on identifying key determinants associated with FCP adoption and implementation. However, due to some concerns about the effects of FCP on patient safety and clinical outcomes, future research is needed to fully explore the effects of FCP on patients boarded in inpatient hallways.

## Conclusion

This study offers a clear picture of the evolution of FCP through the time, the key barriers that impact FCP adoption and implementation, and practical recommendations to overcome those barriers. Our findings show that the key determinants of successful adoption and implementation of FCP are collaborating with inpatient nursing, achieving consensus about the criteria for activation of each level and actions in each level, complying with external regulations and policies, modifying the electronic health records system, restructuring the inpatient units to have adequate staffing and resources, and gaining hospital leaders’ support. Finally, our findings suggest that FCP adoption and implementation is challenging and requires a dedicated multidisciplinary, hospital-wide team with executive authority to adopt, adapt, and execute an implementation plan that will effectively reduce ED crowding and improve hospital-wide patient care.

## Additional files

Additional file 1: Interview guide. (DOCX 23 kb)

Additional file 2: CFIR CODEBOOK^1^. (DOCX 32 kb)

## Data Availability

The data used and/or analyzed during the current study are available from the corresponding author on reasonable request and subject to IRB guidelines.

## Abbreviations

ACEP: American College of Emergency Physicians
CFIR: Consolidated Framework for Implementation Research
DRH: Division of Reproductive Health
EBPs: Evidence-based practices
EHR: Electronic health records
FCP: Full capacity protocol
