# Supplementary material for: What is full capacity protocol, and how is it implemented successfully?
Source: Implement Sci. 2019 Jul 18;14:73. doi: 10.1186/s13012-019-0925-z (PMC6637572; doi:10.1186/s13012-019-0925-z)
Supplement: Supplementary file 1 — Interview guide. (DOCX 23 kb) [file 13012_2019_925_MOESM1_ESM.docx]

# APPENDIX A: Interview Guide

## Part I: Innovation Characteristics

- Would you describe FCP in your hospital for me? What are the core elements of it? Do you call it FCP or something else?
- Does FCP have ever modified or even de-implemented? If yes, would you explain?
- Does inpatient boarding is a routine practice in your hospital?
- How does FCP compare to other similar existing programs in your hospital? Any advantages/disadvantages?

## Part II: Inner setting

- What do influential people including administrative or other leaders think about ED crowding problems?
- What do influential individuals including administrative or other leaders think of the FCP? How do attitudes of different leaders vary?
- What steps have been taken to convince the hospital leaders and encourage key individuals to adopt FCP if any?
- How do you think your hospital's culture will affect the implementation of the FCP? Can you describe an example that highlights this?
- Did the key individuals participate in the FCP have the skills, knowledge and resources to do so?
- Since implementing FCP what level of endorsement or support have you seen or heard from hospital leaders? What kind of support have they given you?

## Part III: Outer setting

- What kind of local, state, or national performance measures, policies, regulations, or guidelines influenced the decision to not to adopt FCP/ adopting other interventions?
- What kind of financial or other incentives influenced the decision to/not to adopt the FCP?

## Part IV: Process

- Do you face any resistance against this plan? Who resist the most and why?
- What steps have been taken to engage the key stakeholders and encourage them to commit to using the FCP?
- Which individuals did you target?
- What information did you give them?
- How frequently and how did you communicate with them?
- What is your communication or education strategy for getting the word out about the FCP?
- What materials/modes/venues did you use?
