# Supplementary material for: What is full capacity protocol, and how is it implemented successfully?
Source: Implement Sci. 2019 Jul 18;14:73. doi: 10.1186/s13012-019-0925-z (PMC6637572; doi:10.1186/s13012-019-0925-z)
Supplement: Supplementary file 2 — CFIR CODEBOOK1. (DOCX 32 kb) [file 13012_2019_925_MOESM2_ESM.docx]

**APPENDIX B - CFIR CODEBOOK^1^**

| **Innovation Characteristics** | |
| --- | --- |
| Relative Advantage | **Definition:** Stakeholders’ perception of the advantage of implementing the innovation versus an alternative solution.  **Inclusion Criteria:** Include statements that demonstrate the innovation is better (or worse) than existing programs.  **Exclusion Criteria:** Exclude statements that demonstrate a strong need for the innovation and/or that the current situation is untenable and code for tension for change. |
| **Inner Setting** |  |
| Structural Characteristics | **Definition:** The social architecture, age, maturity, and size of an organization. |
| Culture | **Definition:** Norms, values, and basic assumptions of a given organization.  **Inclusion Criteria:** Inclusion criteria, and potential sub-codes, will depend on the framework or definition used for “culture.” For example, if using the [Competing Values Framework](http://www.implementationscience.com/content/2/1/13/abstract) (CVF), you may include four sub-codes related to the four dimensions of the CVF and code statements regarding one or more of the four dimension in an organization. |
| Leadership Engagement | **Definition:** Commitment, involvement, and accountability of leaders and managers with the implementation of the innovation.  Inclusion Criteria: Include statements regarding the level of engagement of organizational leadership.  **Exclusion Criteria**: Exclude or double code statements regarding leadership engagement to Engaging: [Formally Appointed Internal Implementation Leaders](http://cfirwiki.net/wiki/index.php?title=Formally_Appointed_Internal_Implementation_Leaders) or [Champions](http://cfirwiki.net/wiki/index.php?title=Champions) *if* an organizational leader is also an implementation leader, e.g., if a director of primary care takes the lead in implementing a new treatment guideline. Note that a key characteristic of this Implementation Leader/Champion is that s/he is also an Organizational Leader. |
| Access to Knowledge & Information | **Definition:** Ease of access to digestible information and knowledge about the innovation and how to incorporate it into work tasks.  **Inclusion Criteria:** Include statements related to implementation leaders' and users' access to knowledge and information regarding use of the program, i.e., training on the mechanics of the program.  **Exclusion Criteria:** Exclude statements related to engagement strategies and outcomes, e.g., how key stakeholders became engaged with the innovation and what their role is in implementation, and code to [Engaging](http://cfirwiki.net/wiki/index.php?title=Engaging): Key Stakeholders.  Exclude statements about general networking, communication, and relationships in the organization, such as descriptions of meetings, email groups, or other methods of keeping people connected and informed, and statements related to team formation, quality, and functioning, and code to [Networks & Communications](http://cfirwiki.net/wiki/index.php?title=Networks_%26_Communications). |
| Implementation Climate | **Definition:** The absorptive capacity for change, shared receptivity of involved individuals to an innovation, and the extent to which use of that innovation will be rewarded, supported, and expected within their organization.  **Inclusion Criteria:** Include statements regarding the general level of receptivity to implementing the innovation.  **Exclusion Criteria:** Exclude statements regarding the general level of receptivity that are captured in the sub-codes. |
| **Outer Setting** | |
| External Policy & Incentives | **Definition:** A broad construct that includes external strategies to spread innovations including policy and regulations (governmental or other central entity), external mandates, recommendations and guidelines, pay-for-performance, collaboratives, and public or benchmark reporting.  **Inclusion Criteria**: Include descriptions of external performance measures from the system. |
| **Process** |  |
| Key Stakeholders | **Definition:** Individuals from within the organization that are directly impacted by the innovation, e.g., staff responsible for making referrals to a new program or using a new work process.  **Inclusion Criteria:** Include statements related to engagement strategies and outcomes, e.g., how key stakeholders became engaged with the innovation and what their role is in implementation. Note: Although both strategies and outcomes are coded here, the outcome of efforts to engage staff determines the rating, i.e., if there are repeated attempts to engage key stakeholders that are unsuccessful, the construct receives a negative rating.  **Exclusion Criteria:** Exclude statements related to implementation leaders' and users' access to knowledge and information regarding using the program, i.e., training on the mechanics of the program, and code to [Access to Knowledge & Information](http://cfirwiki.net/wiki/index.php?title=Access_to_Knowledge_%26_Information).  Exclude statements about general networking, communication, and relationships in the organization, such as descriptions of meetings, email groups, or other methods of keeping people connected and informed, and statements related to team formation, quality, and functioning, and code to [Networks & Communications](http://cfirwiki.net/wiki/index.php?title=Networks_%26_Communications). |

**General Coding Rules:**

When two codes are in question for a passage, consider the primary meaning of the passage to assign code; consider what the participant is truly saying. Analysts may wish to err on the side of inclusion or double coding.

1. Adapted from <https://cfirguide.org/>
